# Clinical response to isotretinoin and interferon-α of two dogs with cutaneous epitheliotropic T-cell lymphoma: a case report

**DOI:** 10.1186/s12917-018-1710-y

**Published:** 2018-12-04

**Authors:** Ga-Won Lee, Su-Bin Song, Min-Hee Kang, Hee-Myung Park

**Affiliations:** Department of Veterinary Internal Medicine, College of Veterinary Medicine, Konkuk University, # 1 Hwayang-dong, Gwang-jin-gu, Seoul, 143-701 South Korea

**Keywords:** Canine, Cutaneous T-cell lymphoma, Retinoids, Interferon-α

## Abstract

**Background:**

There is no specific therapy for cutaneous epitheliotropic T-cell lymphoma (CETL). The administration of retinoids in conjunction with interferon-α (IFN-α) in CETL has not been reported in dogs.

**Case presentation:**

Two dogs (Shih tzu and Miniature pinscher) presented with multiple nodular skin lesions. Histopathological examination revealed diffuse infiltrations of lymphocytes in the epidermis and dermis, with a CD3-positive immunophenotypic profile. Based on the clinical and histopathological examination, CETL was diagnosed. Both dogs were treated with isotretinoin in combination with IFN-α and showed clinical improvement with complete or partial remission. The disease in these dogs was well-controlled for more than 264 days of overall median survival time without any additional clinical signs after initiation of the treatment. In both the cases, the dogs were followed up for 27 months, and 10 months without any evidence of recurrence or metastasis, respectively.

**Conclusions:**

We describe the clinical efficacy of isotretinoin combined with IFN-α in 2 dogs with CETL. Long-term management with isotretinoin combined with IFN-α was effective in treating CETL in these cases.

## Background

Canine cutaneous lymphoma is a relatively uncommon form of lymphoma with an incidence of 3–8% of lymphoma, and 1% canine skin tumors [1–3]. Cutaneous epitheliotropic T-cell lymphoma (CETL) is well characterized by lesions such as exfoliate erythroderma with pruritic erythema, scaling, mucocutaneous erythema, depigmentation, ulceration, and solitary or multiple nodules or plaques [4]. In CETL, cytology samples can reveal large round cells with a high nucleo-cytoplasm ratio and prominent nucleoli with histological presence of cutaneous infiltration of malignant T lymphoid cells, especially in the epidermis and dermis [4]. In humans, it progresses through multiple stages starting with patches (patch stage), followed by plaques of variable thickness (plaque stage), and finally transforms into the tumor stage [5]; in contrast to humans, these clinical manifestations may be seen in a random order in canine CETL [4]. The diagnosis of CETL is confirmed by histopathological examination of skin and immunophenotype for T-cells [2–4].

Treatment for epitheliotropic lymphoma is not curative but palliative in both canine and human patients [4, 6]. There is no standard therapy for canine CETL, but variable treatment options have been developed. Systemic therapies including glucocorticoids, retinoids, interferons, dacarbazine, lomustine (CCNU, 1-(2-Chloroethyl)-3-cyclohexyl-1-nitrosourea) for canine CETL have been reported, and all these reported therapies reflect clinical improvement for 3 to 15 months [4, 7–13]. Other choices include surgical resection, radiation therapy and supportive therapy [8, 9].

The numerous applicable protocols showed the variable and frequently short-lived responses, but among them, chemotherapy is recommended as a common first choice for treatment of CETL in dogs [9], and the most promising protocols include lomustine (CCNU) with 82% overall response rate and the median survival time of 6 months [3, 6, 11, 14]. In general, the prognosis of CETL is poor with variable clinical manifestations from indolent disease to aggressive and progressive disease in dogs [2, 3, 7]. Especially, the presence of multiple lesions can be associated with shorter median survival time than time of mucocutaneous form and the presence of a single lesion [6].

This report describes the clinical response of 2 dogs with CETL that were treated with isotretinoin in combination with interferon-α (IFN-α). To the best of the authors’ knowledge, no study has shown clinical features and outcomes of canine CETL treated with isotretinoin and IFN-α.

## Case presentation

### Case 1

**Fig. 1:**
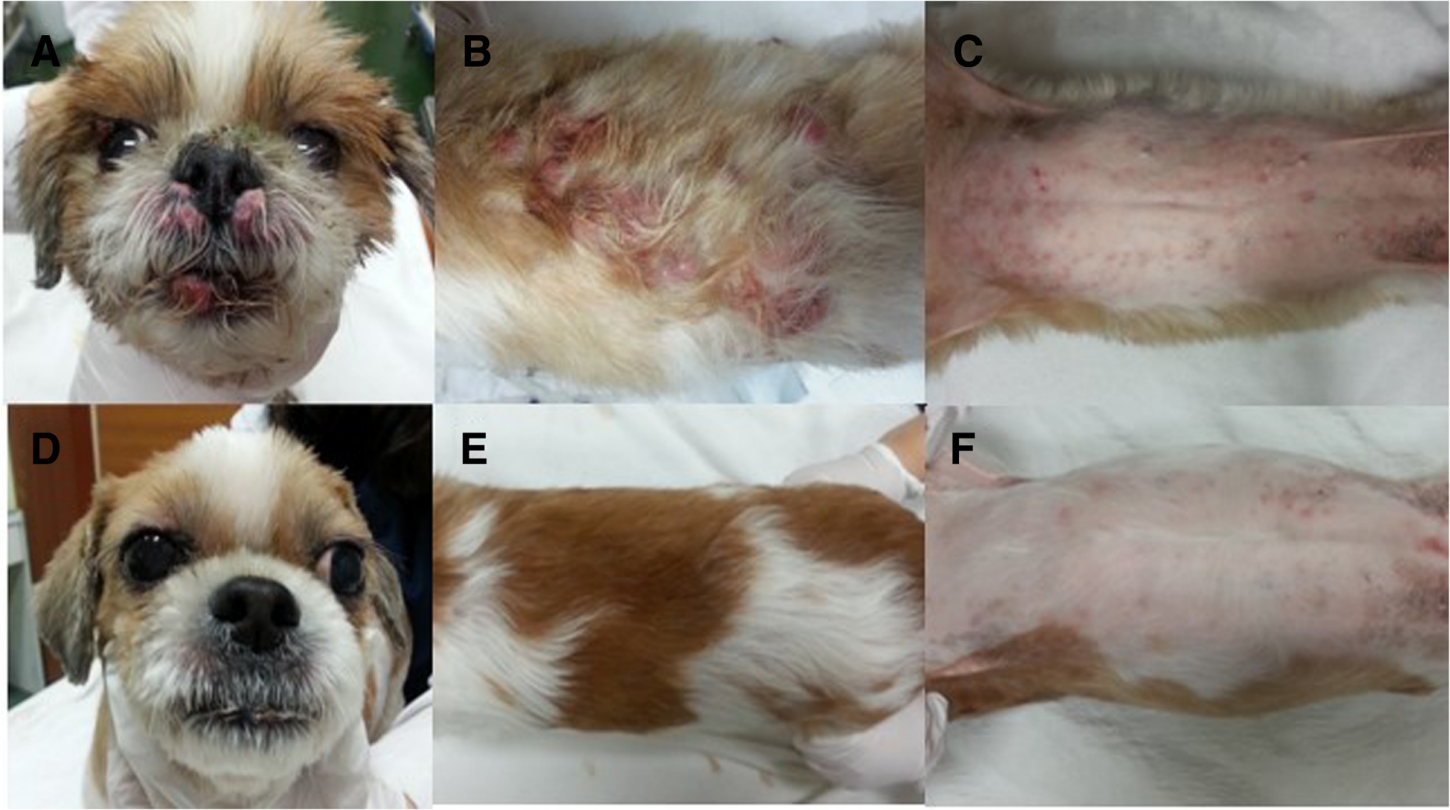
General appearance of epitheliotropic lymphoma in a Shih tzu dog (case 1). Multiple erythematous nodules were marked on the face (**a**) and the dorsum (**b**), and multiple papules detected on the ventral skin lesion (**c**). Three months after initiation of isotretinoin in conjunction with IFN-α shows complete disappearance of nodules and complete remission (**d**, **e**, and **f**)

A 12-year-old, spayed, female Shih tzu dog was referred for evaluation of multifocal cutaneous masses. The masses had appeared 7 weeks before visiting the hospital. On skin examination, generalized nodules on the dorsum, flank, tail, ear, eyelid, muzzle and multiple papules in the axillary, inguinal region and ventrum were found (Fig. 1a-c). The nodules were well-demarcated with erythema. No further abnormalities were detected, and superficial lymph nodes were found to be within normal size on palpation. Both hematology and serum chemistry analysis were within normal range.

**Fig. 2:**
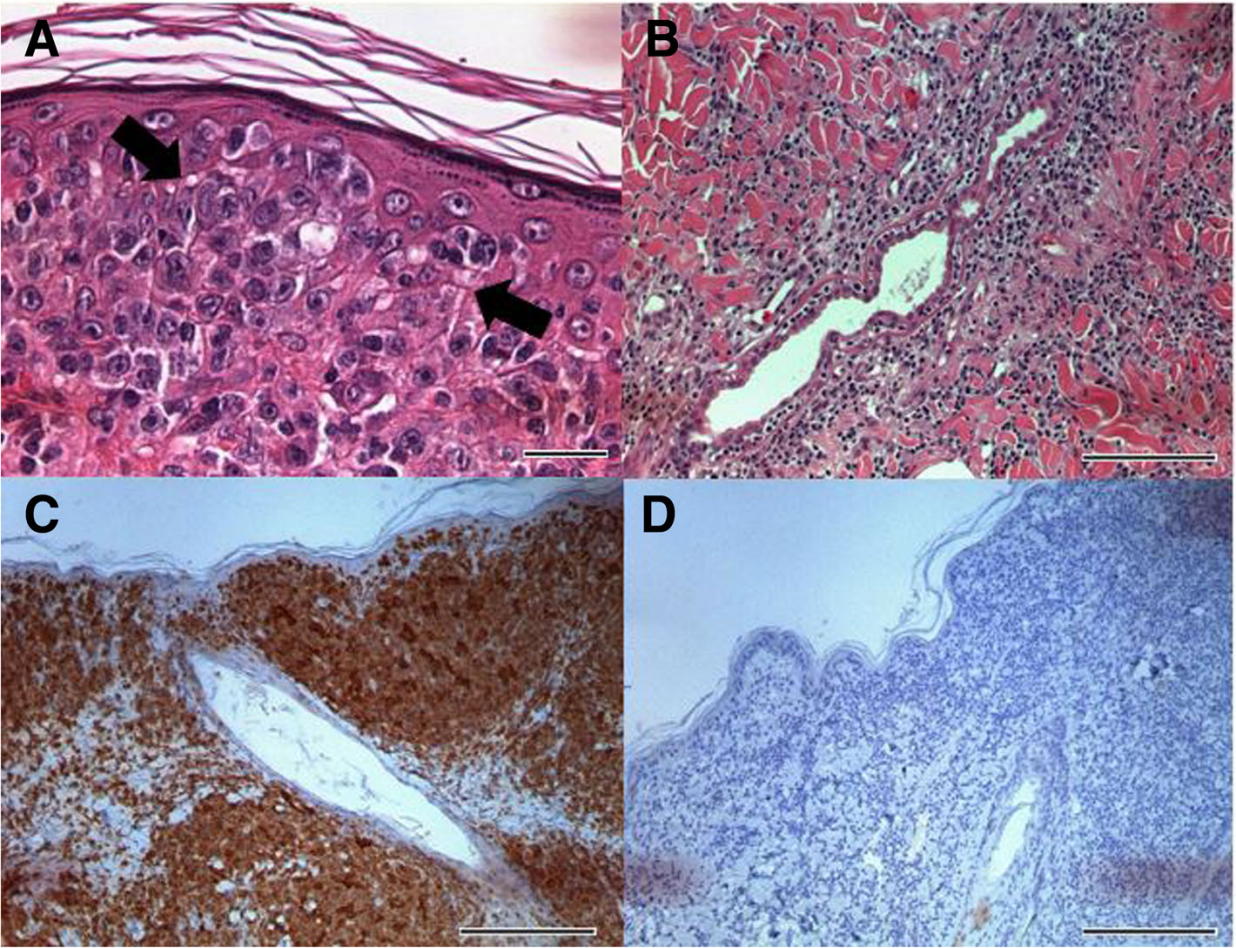
Histopathology of nodules of dorsum and trunk of case 1. Note the tropism of neoplastic lymphocytes in the epidermis (**a**; bar = 20 μm, H&E stain) and apocrine sweat glands (**b**: bar = 100 μm, H&E stain), with formation of Pautrier’s microabscesses (arrows), indicating mycosis fungoides. Immunohistochemical results show numerous CD3 positive (**c**: CD3, bar = 200 μm) & CD79a negative cells (**d**: CD79a, bar = 200 μm), indicating T-cell epitheliotropic lymphoma

Differential diagnoses for the skin lesions included epitheliotropic lymphoma, atypical histiocytoma, cutaneous histiocytosis, plasmacytoma and mast cell tumor. Incisional biopsies of the dorsal skin nodules revealed diffuse infiltrations of lymphocytes in the epidermis and dermis (Fig. 2a-b). Detection of neoplastic lymphocytes observed in the epidermis was consistent with Pautrier’s microabscess (Fig. 2a). Tropism for hair follicles and adnexal glands was observed in the dermis (Fig. 2b). Immunohistochemical results showed numerous CD3 positive (Fig. 2c) and CD79a negative cells (Fig. 2d), which indicated CETL. Treatment was initiated with isotretinoin (2 mg/kg, PO, once daily; Roaccutane, La Roche Pharma, Basel, Switzerland) in combination with IFN-α (1.5 × 10^6^ IU/m^2^, SC, every other day; Roferon-A; La Roche Pharma, Basel, Switzerland).

The dog demonstrated clinical improvement within 4 days following the initiation of the multimodality therapy. The treatment was continued and almost all papules on the abdomen disappeared. There was complete disappearance of the nodules following the 3-month duration of therapy, indicating complete remission (Fig. 1d-f). The treatment discontinued after complete remission, and the case was followed up for 27 months without any evidence of recurrence or metastasis.

### Case 2

**Fig. 3:**
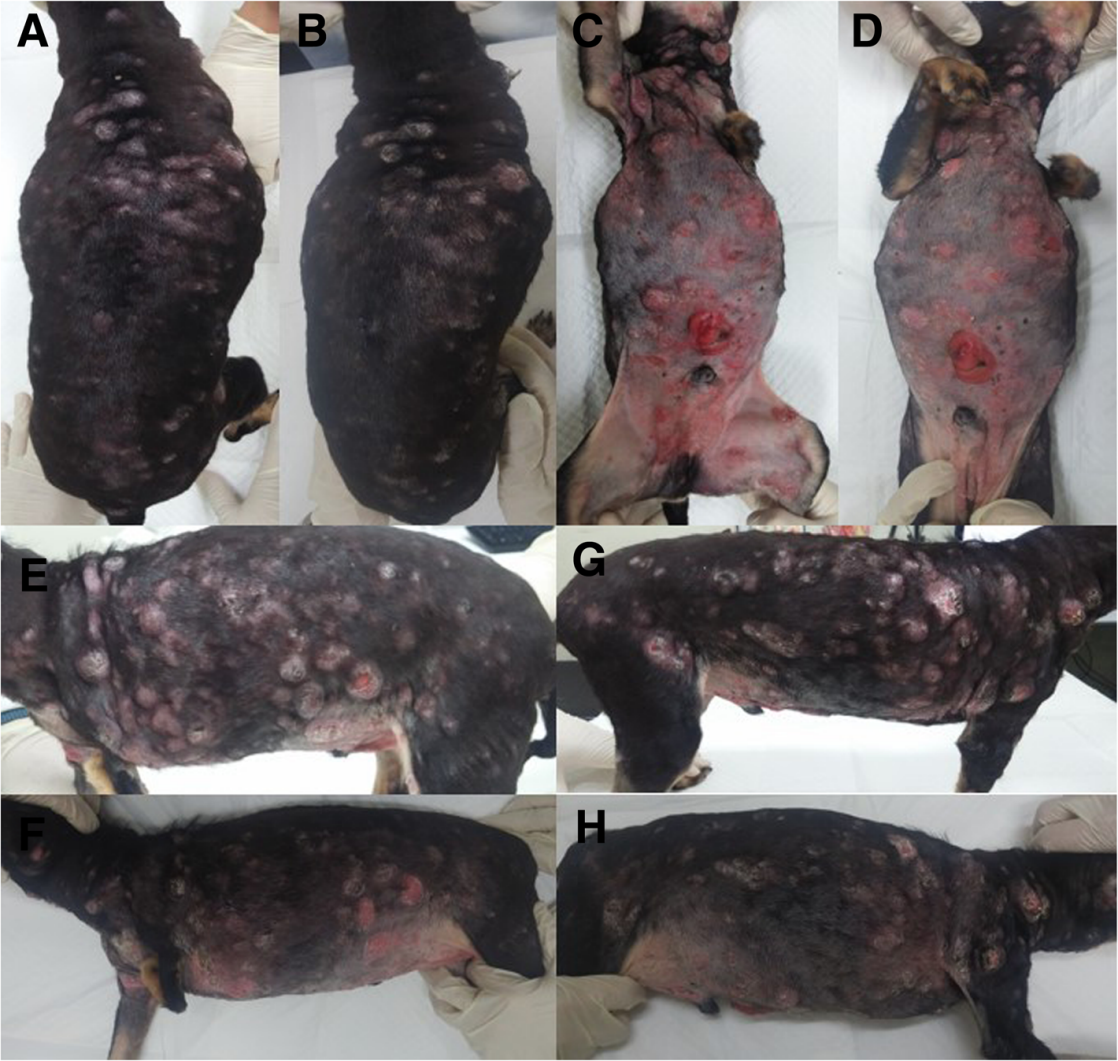
General appearance of epitheliotropic lymphoma in a Miniature pinscher dog (case 2). Generalized ulcerative nodules, characteristic of epitheliotropic lymphoma, were detected at the time of diagnosis (**a**, **c**, **e**, and **g**) and 2 weeks after administration of isotretinoin with IFN-α (**b**, **d**, **f**, and **h**). Skin lesions remarkably improved 2 weeks after multimodality therapy. The improvement of clinical features was well maintained up to 2 months after diagnosis

A 12-year old, castrated, male Miniature pinscher dog presented with history of generalized nodules and a ventral umbilical hernia. The umbilical hernia was first observed and the nodules were disseminated, which worsened over a period of 2 months with gradual increase in size (Fig. 3a, c, e and g). The owner reported that the dog received oral prednisolone and antibiotics for 2 weeks before this referral, but there was no improvement.

**Fig. 4:**
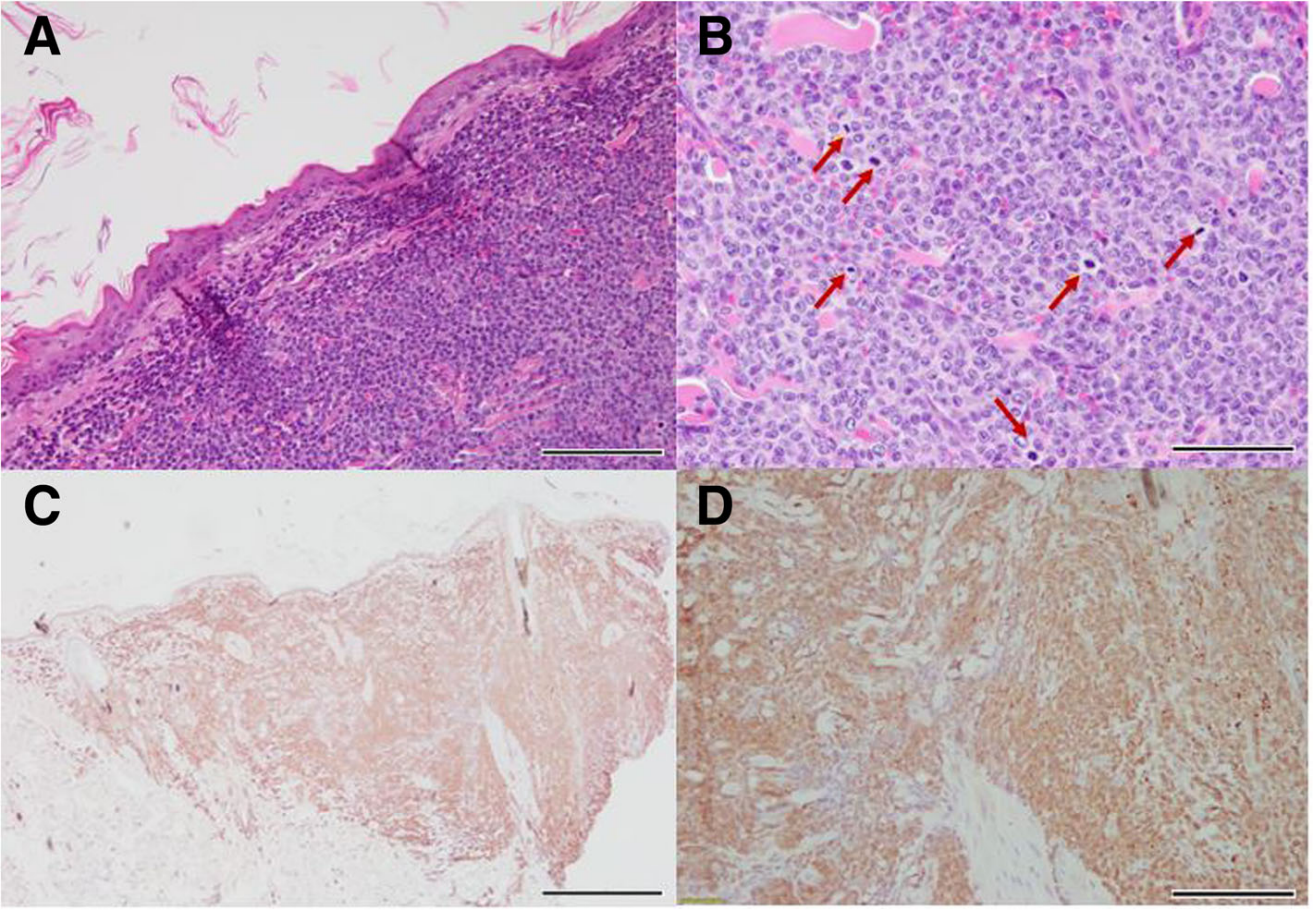
Histopathology of nodules of dorsum and trunk of case 2. Epidermal and dermal infiltration of lymphoid cells with large nuclei and high mitotic activity (red arrows) are shown in A & B (**a**: bar = 100 μm, **b**: bar = 50 μm; H&E stain). The tropism of neoplastic lymphocytes in the epidermis was observed, diagnosed as mycosis fungoides. Immunohistochemistry for CD3 (C & D) reveals infiltration of highly CD3-positive neoplastic lymphocytes in the epidermis and dermis (**c**: bar = 500 μm, **d**: bar = 100 μm)

There was no enlargement of the peripheral lymph nodes on palpation and no other abnormalities were noted on the hematologic or serum biochemical examination. Dermatologic examination revealed generalized ulcerative nodules, erosion, erythema and hyperpigmentation in the dorsum, ventrum, neck and perianal region. Multiple, 6 mm, punch skin biopsies were performed of the nodular lesions for histopathological evaluation. Impression cytology showed numerous neutrophils and phagocytized cocci bacteria and the fine needle aspiration biopsy revealed cluster of intermediate to large lymphocytes with multinuclear cells. Histopathological results showed proliferation of numerous round cells and mitotic figures were identified in the epidermis and dermis (Fig. 4a-b). Through immunohistochemical staining, numerous round cells were CD3 positive, confirming T cell origin (Fig. 4c-d). Based on the skin lesions and histological findings, the dog was diagnosed with CETL.

The dog was initially treated with retinoic acid (1.5 mg/kg, PO, once daily; Roaccutane; La Roche Pharma, Basel, Switzerland) and IFN-α (2 × 10^6^ IU/m^2^, SC, every other day; Roferon-A; La Roche Pharma, Basel, Switzerland). 2 weeks later, there was marked improvement in the skin condition showing approximately more than 50% regression in the size of the nodules, termed as partial remission (Fig. 3b, d, f and h). The dog was treated for 2 months and clinical condition was well controlled without any other side effects. The case was followed up for 10 months and there was no evidence of recurrence or metastasis.

## Discussion

Cutaneous T-cell lymphoma can be classified as non-epitheliotropic or epitheliotropic. Epitheliotropic lymphoma is characterized by an infiltration of neoplastic lymphocytes with a high tropism for the epidermis [15]. CETL, a T-cell neoplasia can be histologically classified into mycosis fungoides, Pagetoid reticulosis and Sézary syndrome [15, 16]. In both of the cases, a striking tropism for hair follicles and epitrichial sweat glands was revealed, indicating canine mycosis fungoides, unlike what is seen in humans [16]. Sézary syndrome, which has a significant population of circulating atypical T-lymphocytes in blood and a more aggressive course than mycosis fungoides was ruled out after blood smear. Pagetoid reticulosis shows pure tropism in epithelium, so it was ruled out based on invasion of dermis by neoplastic lymphocytes in both cases [15, 17]. Mycosis fungoides is the most common form of CETL in both dogs and humans, but progression is more rapid in dogs than in humans [16].

It is known that generalized CETL has a worse prognosis than multicentric lymphoma [1, 2, 4], and as it is in progress, response to treatment would be reduced [5]. Therefore, appropriate implement of effective treatment with definitive diagnosis through the biopsy of suspected lesions for CETL is emphasized. Advanced clinical stage in canine lymphoma is associated with poor prognostic factors, and canine CETL has non-lymphoid organ involvement and is included in stage V according to WHO (World Health Organization) clinical staging for domestic animals with lymphoma. Even though the cases of this report are included in advanced clinical stage, which is the clinical stage V-a, the clinical response to the combination treatment of isotretinoin and IFN-α was quite good with long follow-up period of 27 and 10 months in each case.

Retinoids control cell growth and differentiation in a variety of human tumors by reducing the expression of Bcl-2 protein and upregulating bax proteins [5]. Moreover, they inhibit cell growth by arresting cells in the G1 phase or inducing apoptosis, that has an anti-carcinogenic effect on T-cell lymphoma cell lines [4, 5, 17–20]. Retinoids are used effectively for the treatment of promyelocytic leukemia, cutaneous lymphoma, lung and thyroid carcinomas, and glioblastomas in humans [20], as well as CETL in dogs [19]. Isotretinoin is an especially well-tolerated, oral drug that provides good alleviation of clinical signs associated with CETL, but the exact mechanism of action is unclear [5, 17].

IFN is known to have antitumor activity against malignant lymphomas, particularly the indolent B-cell lymphomas and CETL in humans [20]. However, the effects of IFN-α on the different stages of CETL remain controversial [21–23]. IFN-α is also useful in the palliative management of advanced or refractory CETL in canines as well as humans [4].

The adverse effects of retinoids include panting, corneal lipid deposits, dry cough and keratoconjunctivitis sicca [13, 24], and myelosuppression and vitamin A toxicity like hepatotoxicity were reported as the side effects of IFN-α in dogs [15, 25]. The adverse effects of both drugs are non-overlapping and well tolerated, and the possibility of toxicity is minimal in dogs [13, 15, 25] and humans [20]. The major disadvantages of retinoids alone are the time lapse between initiation of therapy and clinical response and the relatively high cost of the drug [15]. However, in combination with IFN-α, the time lapse could be shortened and the cost of the treatment with isotretinoin in combination with IFN-α is affordable compared to chemotherapy in practice. In both the cases, the clinical improvement was observed within 2 weeks and there were no side effects after the administration of the drugs and the clinical signs improved, increasing the quality of life.

Synergism between IFN-α and retinoids was revealed in vitro, and the combination could derive higher levels of IFN-stimulated genes [20]. Moreover, IFN-α facilitated retinoid-induced differentiation, whereas, retinoids otherwise increase the anti-proliferative activity of IFNs [20]. In humans, retinoids combined with IFN-α are effective in treating squamous cell carcinoma and lymphomas, especially CETL, with a response rate of 33% [20]. Retinoid stimulation of Th1 activity by IL-12 production and inhibition of Th2 activity by IFN-α, shows synergistic effects [17]; it is speculated that focusing on cellular immunity is suitable for the treatment of CETL and resulted in favorable response and outcome in both the cases of this report.

## Conclusions

In conclusion, this report describes the cases of 2 dogs with CETL treated successfully with isotretinoin in conjunction with IFN-α, with an achievement of long-term clinical remission. To the best of the authors’ knowledge, no study in veterinary medicine has shown clinical features and response of CETL in dogs treated with this multimodality therapy till date. This report suggests that IFN-α in conjunction with isotretinoin may be useful for management of canine CETL, which has no specific therapy and is frequently refractory to conventional chemotherapy. It is supposed that this combination, without systemic chemotherapy, could have effective antitumor activity in canine CETL and be well tolerated in dogs with advanced CETL without any serious side effects.

## Abbreviations

CETL: Cutaneous epitheliotropic T-cell lymphoma
IFN-α: Interferon-α
